# Persistent and heterogeneous socioeconomic inequalities in women’s malnutrition across 70 low- and middle-income countries

**DOI:** 10.3389/fpubh.2026.1910404

**Published:** 2026-08-20

**Authors:** Ling Yang, Si-Qi Jin, Muhammad Riaz, Najm Ur Rahman

**Affiliations:** 1 Department of Geriatrics, Shanghai Fourth People's Hospital Affiliated to Tongji University, Shanghai, China; 2 School of Exercise and Health, Shanghai University of Sport, Shanghai, China; 3 Department of Pharmacy, Shaheed Benazir Bhutto University, Sheringal, Pakistan; 4 Department of Pharmaceutical Chemistry, Faculty of Pharmacy, Universiti Malaya, Kuala Lumpur, Malaysia

**Keywords:** demographic and health surveys, health inequality, malnutrition, nutrition transition, slope index of inequality, socioeconomic gradients, women’s health

## Abstract

**Background:**

Socioeconomic inequalities in malnutrition remain a major public health concern in low- and middle-income countries (LMICs). While patterns of undernutrition and excess weight are well documented, less is known about how their distribution across socioeconomic groups has changed over time. We aimed to examine cross-country patterns and descriptive temporal patterns of socioeconomic inequalities in women’s nutritional status across LMICs.

**Methods and findings:**

We conducted a repeated cross-sectional analysis using the World Health Organization’s Health Inequality Data Repository, which includes Demographic and Health Survey data from 70 LMICs between 1991 and 2023. National estimates of anemia, thinness, overweight, obesity, and combined overweight/obesity were analyzed by wealth and place of residence; education-based analysis was treated as exploratory due to limited coverage. Wealth-related inequality was measured using the slope index of inequality (SII). Year-specific summaries were calculated as unweighted medians across the countries observed in each calendar year, without interpolation. In country-specific latest surveys, the median wealth-related SII was as follows: −6.2 percentage points for anemia, −5.6 for thinness, +14.0 for overweight, +13.2 for obesity, and +25.0 for combined overweight/obesity. Among the countries with repeated observations, overweight, obesity, and combined overweight/obesity remained pro-advantaged in 89.7, 84.5, and 87.9% of country series, respectively; reversals in these trends occurred in only 6.9–8.6% of country series. Sensitivity analyses conducted on all available survey rounds generally found no clear linear change in the magnitude of inequality, although strengthening was more frequent for obesity. Overall, comparisons of repeated country observations showed a mixture of persistent, attenuating, strengthening, and reversing inequality patterns. Mixed-effects models supported overall differences across survey periods but highlighted substantial variability between countries.

**Conclusion:**

Socioeconomic inequalities in women’s malnutrition were predominantly persistent in direction but heterogeneous in magnitude across countries. Deficiency-related outcomes remained concentrated among disadvantaged women, whereas overweight and obesity generally remained concentrated among advantaged women, with relatively uncommon reversal. These repeated cross-sectional findings support continuous monitoring of both nutritional burden and its socioeconomic distribution but do not directly identify the mechanisms responsible for the observed patterns.

## 1 Introduction

Inequalities in nutritional status have long been recognized as a defining feature of population health in low- and middle-income countries (LMICs). Traditionally, the burden of malnutrition, particularly undernutrition and micronutrient deficiencies, has been concentrated among socioeconomically disadvantaged groups (1, 2). This pattern is well documented and, in many settings, persists despite overall improvements in health and living conditions (3, 4). At the same time, many LMICs are undergoing broader nutritional and epidemiological transitions. Alongside persistent undernutrition, the prevalence of overweight and obesity has also increased, often at a rapid pace (5, 6). This coexistence of deficiency and excess has been described as the “double burden” of malnutrition (6, 7). However, while changes in overall prevalence are widely reported, less attention has been given to how the distribution of these outcomes across socioeconomic groups has evolved over time (8).

Earlier studies have often suggested that overweight and obesity are initially more common among wealthier or more educated populations in LMICs, contrasting with patterns observed in high-income countries (9, 10). However, there is some indication that this gradient may shift over time, with excess weight increasingly affecting disadvantaged groups in certain contexts (6, 8). Evidence for such transitions remains mixed, and in many cases, is limited to specific regions, single countries, or individual outcomes (11, 12).

These changes can be interpreted in the framework of nutrition transition. In the early stages of transition, women with higher income or education, especially those in urban settings, may be more exposed to energy-dense diets, sedentary work, and motorized transportation, resulting in an initially pro-advantaged gradient in overweight and obesity. As food systems and living patterns change, inexpensive energy-dense foods and sedentary lifestyles may become more common across socioeconomic groups. Meanwhile, more advantaged women may be better positioned to access healthier foods, use health information, and engage in leisure-time physical activities. Thus, the socioeconomic gradient may be blunted and, in some settings, ultimately reversed. This process is likely not uniform, as urbanization, food environments, gender norms, economic development, and persistent food insecurity vary widely across LMICs (5, 6, 8–10). Thus, the double burden should be understood not only as the co-existence of undernutrition and excess weight but also as a potentially changing social distribution of these outcomes.

A related issue is that inequalities are often studied in isolation, focusing on a single indicator or a single dimension of socioeconomic position (13, 14). This approach risks missing broader patterns, especially where different forms of malnutrition coexist and possibly follow different social gradients (1, 6). Moreover, cross-country comparisons are often hampered by different data sources, measurement, and analytical approaches (15, 16).

The World Health Organization’s Health Inequality Data Repository presents a unique chance to address some of these gaps. This allows for a more systematic analysis of inequality patterns across a range of nutritional outcomes and socioeconomic dimensions by combining standardized, disaggregated estimates from multiple countries and survey years (17).

Nevertheless, the availability of harmonized data does not, by itself, address the substantive evidence gap. Previous multi-country studies have established the coexistence of undernutrition and excess weight and have frequently documented a pro-advantaged pattern of overweight and obesity. However, less is known about whether socioeconomic gradients in deficiency- and excess-related outcomes persist, weaken, or reverse within countries over time, or whether these patterns differ across dimensions of socioeconomic position (7, 11, 12).

In this study, we examine socioeconomic inequalities in women’s nutritional status across 70 LMICs. Wealth and place of residence constitute the primary dimensions of inequality, while education is examined only in an exploratory manner due to limited complete subgroup coverage. We describe national prevalence and socioeconomic inequality separately and assess whether country-specific wealth gradients persist, attenuate, strengthen, emerge, or reverse between available survey observations. Rather than assuming a uniform transition, we approach this analysis with the expectation that inequality patterns may be heterogeneous and, at times, contradictory. Understanding these patterns may be important for designing policies that address not only the overall burden of malnutrition but also its unequal distribution across populations. The present study advances previous multi-country evidence by jointly examining deficiency- and excess-related outcomes, quantifying both the direction and magnitude of inequality, and evaluating country-specific earliest-to-latest patterns, together with sensitivity analyses using all available survey rounds.

## 2 Methods

### 2.1 Study design and data source

We conducted a repeated cross-sectional analysis using data from the World Health Organization (WHO) Health Inequality Data Repository (17). Specifically, we used the adult health and nutrition dataset derived from Demographic and Health Surveys (DHS), which provides nationally representative, disaggregated estimates across multiple countries and time periods (18). The analysis included 70 low- and middle-income countries (LMICs) with available data between 1991 and 2023. Countries were classified using the historical World Bank income classification corresponding to the year of their first analytically eligible survey. Countries classified as low-, lower-middle-, or upper-middle-income in that year were eligible. The static 2025 income classification included in the source database was retained only for descriptive purposes and was not used to determine eligibility. The final sample included 42 low-, 25 lower middle-, and 3 upper middle-income countries at their first eligible survey. The country list is provided in Supplementary Table S3.

### 2.2 Study population and outcomes

The study focused on women of reproductive age, consistent with the Demographic and Health Survey (DHS) design (18, 19). We examined five nutritional indicators: anemia, thinness, overweight, obesity, and combined overweight/obesity. Thinness was defined as a BMI of <18.5 kg/m^2^, overweight as a BMI between 25.0 and 29.9 kg/m^2^, obesity as a BMI of ≥30.0 kg/m^2^, and combined overweight/obesity as BMI of ≥25.0 kg/m^2^ (20, 21). Indicators were selected for their consistent definitions and availability across countries and survey years to ensure comparability. Only female-specific indicators were included to maintain consistency with the study population. A full list of available indicators and their inclusion status is provided in Supplementary Table S4. Indicators not directly related to nutritional status or those with limited cross-country comparability were excluded from the primary analysis.

### 2.3 Dimensions of inequality

Wealth and place of residence were identified as the primary dimensions of inequality. Wealth was treated as an ordered dimension, while residence was analyzed in terms of the urban–rural contrast. Education was retained only as an exploratory ordered dimension because complete education panels were available for very few countries and survey years. For ordered dimensions, subgroups were ranked from the most disadvantaged to the most advantaged based on their inherent order. This ranking was used to construct relative socioeconomic positions for regression-based inequality measures, following standard approaches for analyzing health inequalities (22).

### 2.4 Data processing and inclusion criteria

The source dataset was the adult health and nutrition dataset from the WHO Health Inequality Data Repository, derived from demographic and health surveys. The fields extracted for data processing and analysis were country, survey year, data source, indicator abbreviation and name, inequality dimension, subgroup, prevalence estimate, standard error, lower and upper confidence limits, subgroup population, data-quality flag, national setting average, ISO3 code, indicator direction and scale, ordered-dimension indicator, subgroup order, reference subgroup, WHO region, World Bank income classification, repository registration and update fields, and dataset identifier.

Text fields were standardized by removing leading, trailing, and repeated internal spaces, while survey year, prevalence estimates, standard errors, confidence limits, subgroup population, national averages, subgroup order, and reference-subgroup fields were converted to numeric format. Records were retained only when country, survey year, indicator, inequality dimension, subgroup, and prevalence estimate were available.

The dataset was then restricted to female-specific estimates for anemia, thinness, overweight, obesity, and combined overweight/obesity. Wealth and place of residence were treated as the primary inequality dimensions, while education was retained as an exploratory dimension because complete education subgroup panels were sparse. Indicator names and inequality dimensions were mapped to standardized analytical labels. Residence subgroups were standardized as urban and rural, and ordered wealth and education subgroups were arranged using the subgroup-order field supplied by the repository.

Eligibility as an LMIC was determined using the historical World Bank income classification corresponding to each country’s first analytically eligible survey year. Countries classified as low-, lower-middle-, or upper-middle-income at that time were retained. The static 2025 income classification included in the source dataset was retained only for descriptive auditing and was not used to define eligibility.

To ensure the comparability of inequality estimates across countries and survey years, analyses were restricted to complete country-year-indicator-dimension panels. A panel was defined by a unique combination of country, survey year, nutritional indicator, and inequality dimension. Wealth-based panels required all five wealth quintiles, education-based panels required all four education categories, and residence-based panels required both urban and rural subgroups. Completeness was assessed by comparing the number of distinct observed subgroups with the expected number for the relevant inequality dimension. Panels that did not contain exactly the required number of subgroups were excluded.

The completeness filter was based on subgroup availability and did not require subgroup population values to be present on every record. For ordered dimensions, subgroup population values were used to calculate subgroup proportions and population-weighted RidiT ranks. When at least some valid population information was available, missing or negative subgroup population values were assigned a weight of zero. When all subgroup population values were missing, or when their total was non-positive, equal subgroup weights were used. The resulting weighting method was recorded for each inequality estimate.

Observations with incomplete subgroup information were excluded because partial subgroup panels can bias regression-based inequality measures and reduce comparability across populations (22). The source dataset contained 77,355 subgroup-level records. After cleaning and restriction to the prespecified female nutrition indicators and inequality dimensions, 13,419 records remained before subgroup-completeness assessment. Strict completeness filtering resulted in 7,306 subgroup-level records. These represented 1,058 unique country-year-indicator panels and 2,082 country-year-indicator-dimension inequality estimates.

All 1,038 candidate wealth panels were complete. For residence, 1,030 of 1,042 candidate panels were complete. In contrast, only 14 of the 1,062 candidate education panels contained all required education categories. The complete education observations were limited to Armenia and Kyrgyzstan and were therefore analyzed only as exploratory evidence of repository coverage rather than as representative multi-country estimates.

This approach prioritizes the internal comparability of inequality estimates across countries and survey years, although at the cost of a reduced analytical sample. A summary of the included indicators, countries, and time coverage is presented in Table 1. Subgroup completeness before and after applying complete-panel criteria is detailed in Supplementary Table S2.

**Table 1:** Overview of included indicators, countries, and time coverage.

Indicator	Subgroup rows	Country-year-indicator panels	Countries	Years
Anemia	939	133	56	2000–2023
Thinness	1,597	232	70	1991–2023
Overweight	1,590	231	70	1991–2023
Obesity	1,590	231	70	1991–2023
Overweight/obesity	1,590	231	70	1991–2023

### 2.5 Measures of inequality

Absolute differences and ratios compared the most advantaged and most disadvantaged subgroups and therefore represent simple extreme-group contrasts. For wealth, SII incorporated all ordered subgroups. Population-weighted RidiT ranks were calculated as the cumulative subgroup population proportion minus one-half of that proportion. SII was estimated using weighted linear regression of subgroup prevalence on RidiT rank, with subgroup population used as the analytical weight. The SII coefficient represents the predicted absolute prevalence difference, in percentage points, between the theoretical extremes of socioeconomic distribution. Positive values indicate higher prevalence among advantaged women, whereas negative values indicate higher prevalence among disadvantaged women (22, 23). The structure of inequality dimensions and subgroup ordering is summarized in Supplementary Table S1.

### 2.6 Trend analysis and pooled summaries

Inequality measures were calculated separately for each country-year-indicator combination. Because DHS surveys were conducted in different calendar years, the data formed unbalanced repeated cross-sectional series. No annual interpolation was performed. For descriptive calendar-year summaries, unweighted medians and interquartile ranges were calculated across countries observed in each year. Each country therefore contributed equally within a calendar year, although countries with more survey rounds contributed observations to more calendar years. The countries contributing to each yearly estimate, along with their numbers, were reported separately.

### 2.7 Classification of inequality trajectories

Country-specific wealth-related inequality patterns were classified by comparing the earliest and latest available SII estimates among countries with at least two eligible survey rounds. To avoid interpreting very small gradients as meaningful inequality, SII values between −1 and +1 percentage points were classified as near-null.

Classification was applied sequentially. First, transitions involving near-null values were identified. Movement from a near-null SII to a positive or negative non-null SII was classified as emergence, whereas movement from a non-null SII to the near-null range was classified as attenuation to near-null. Second, among non-null estimates, a change in sign between positive and negative SII values was classified as reversal. Finally, for observations remaining on the same side of zero, the proportional change in the absolute magnitude of SII was calculated as (|SII_latest_| − |SII_earliest_|)/|SII_earliest_|. A reduction of at least 25% in the absolute magnitude of SII was classified as attenuation, an increase of at least 25% as strengthening, and intermediate changes as persistence. Direction-specific labels (pro-advantaged or pro-disadvantaged) were retained throughout the classification.

Because earliest-to-latest comparisons do not capture intermediate fluctuations, a prespecified sensitivity analysis was conducted among country-indicator series with at least three survey rounds. All available wealth-related SII observations were included in linear regression models of both signed SII and absolute SII against a calendar year. The regression of signed SII evaluated the direction of change, whereas the regression of absolute SII evaluated changes in inequality magnitude irrespective of direction. Country series were classified as showing evidence of attenuation, strengthening, or no clear linear change in magnitude based on the estimated slope and its statistical significance. These analyses were interpreted as exploratory because the follow-up duration and the number and spacing of survey rounds differed across countries (24).

### 2.8 Statistical analysis

Analyses were conducted in R (25). The unit of analysis for the mixed-effects models was country-year-subgroup observation. Separate models were fitted for each nutritional outcome and for each primary inequality dimension (wealth and place of residence).

The dependent variable was subgroup-specific prevalence expressed as a proportion and transformed into the logit scale. Fixed effects were included calendar year (continuous) and subgroup category (categorical). Country-specific random intercepts and random slopes for calendar years were initially specified to account for repeated observations within countries. Models were estimated by maximum likelihood using the lmer() function from the lme4 package. Model singularity was assessed using the isSingular() function with a tolerance of 1 × 10^−4^. When the random-slope model was singular, it was refitted with only a country-specific random intercept.

Subgroup population values supplied by the WHO Health Inequality Data Repository were used as analytical weights. When subgroup population values were unavailable, or their total was non-positive within a panel, equal subgroup weights were applied during preprocessing. The repository also provided subgroup-level standard errors and confidence intervals; however, these measures of estimate precision were not incorporated into the mixed-effects likelihood because the models were fitted to aggregated prevalence estimates using subgroup population as the analytical weight rather than inverse-variance weighting.

Population-level predicted prevalence curves were generated from the fixed-effects component of each fitted model by excluding country-specific random effects (re.form = NA) and back-transforming predictions from the logit scale to the original prevalence scale.

Because year-by-subgroup interaction terms were not included, these models summarized overall temporal patterns in subgroup prevalence and average subgroup differences but did not directly estimate temporal changes in socioeconomic inequality (26). Changes in inequality were evaluated separately using country-year SII estimates, earliest-to-latest trajectory classifications, and sensitivity analyses that incorporated all available survey rounds. All analyses were performed within a fully reproducible workflow using predefined data-processing, quality-control, statistical-analysis, and figure-generation scripts. The complete R code is provided in Supplementary File S1.

## 3 Results

### 3.1 Study sample and data coverage

The source database contained 77,355 subgroup-level rows. After data cleaning and restriction to the prespecified female nutritional indicators and inequality dimensions, 13,419 rows remained before completeness assessment. Strict completeness filtering produced 7,306 subgroup rows, representing 1,058 country-year-indicator panels and 2,082 country-year-indicator-dimension inequality estimates. The final sample included 70 historically eligible LMICs. Complete wealth data were available for 1,038 of 1,038 candidate panels (100%). Residence data were completed for 1,030 of 1,042 panels (98.85%). Education data were complete for only 14 of 1,062 panels (1.32%), representing Armenia and Kyrgyzstan in 2000, 2005, and 2012. Education-based results were therefore treated as exploratory and were not interpreted as representative of multi-country temporal trajectories.

### 3.2 Global patterns in national prevalence over time

**Figure 1:**
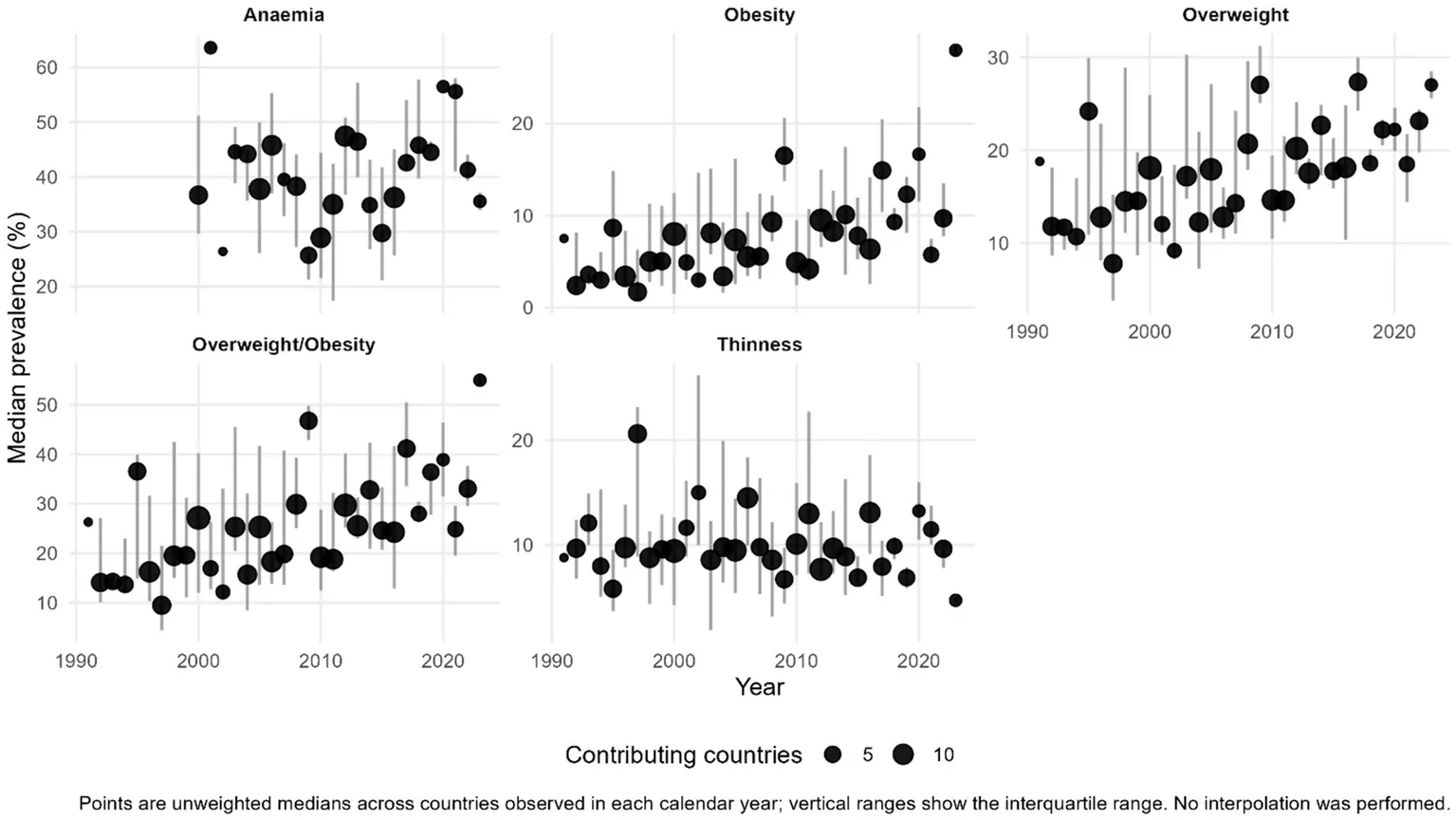
Year-specific median national prevalence of nutritional outcomes. Points represent unweighted medians across countries observed in each calendar year, vertical ranges represent interquartile ranges, and point size indicates the number of contributing countries. No interpolation was performed; therefore, changes between years may reflect both temporal change and changing country composition.

Year-specific pooled prevalence estimates varied substantially because both the countries represented and the number of countries differed across calendar years. Descriptive annual summaries therefore did not represent a fixed-country longitudinal series. Model-based analyses indicated an overall increase in overweight, obesity, and combined overweight/obesity and a decline in thinness, whereas the temporal coefficient for anemia was less precise. Considerable between-country variation remained (Figure 1).

Despite these overall trends, substantial variability remains across countries at all time points. Some country series showed large increases in obesity and overweight across available survey observations, whereas others showed more gradual changes or relative stability. Similarly, while declines in thinness were observed in many settings, the pace and extent of change differed considerably. These findings indicate that, although broad shifts in nutritional burden are evident across LMICs, underlying country-level trajectories remain diverse.

### 3.3 Socioeconomic distribution of nutritional outcomes

Nutritional outcomes across countries were arrayed along distinct contrasting patterns of socioeconomic status. Deficiency-related conditions were consistently more prevalent in disadvantaged groups, particularly anemia and thinness in lower wealth and rural groups.

A different pattern was observed for excess-related outcomes, particularly in the survey’s earlier periods. Overweight and obesity were more prevalent among the more advantaged populations, including the higher wealth quintiles and those living in the urban setting. These patterns were not entirely consistent across countries or over time. The distribution of overweight and obesity was more variable, both in magnitude and in some settings, direction, while disadvantage-associated nutritional deficiency remained relatively stable. These findings suggest that the burdens of deficiency and excess have different socioeconomic gradients and provide a baseline against which changes in inequality over time can be interpreted.

### 3.4 Evolution of wealth-related inequality over time

**Figure 2:**
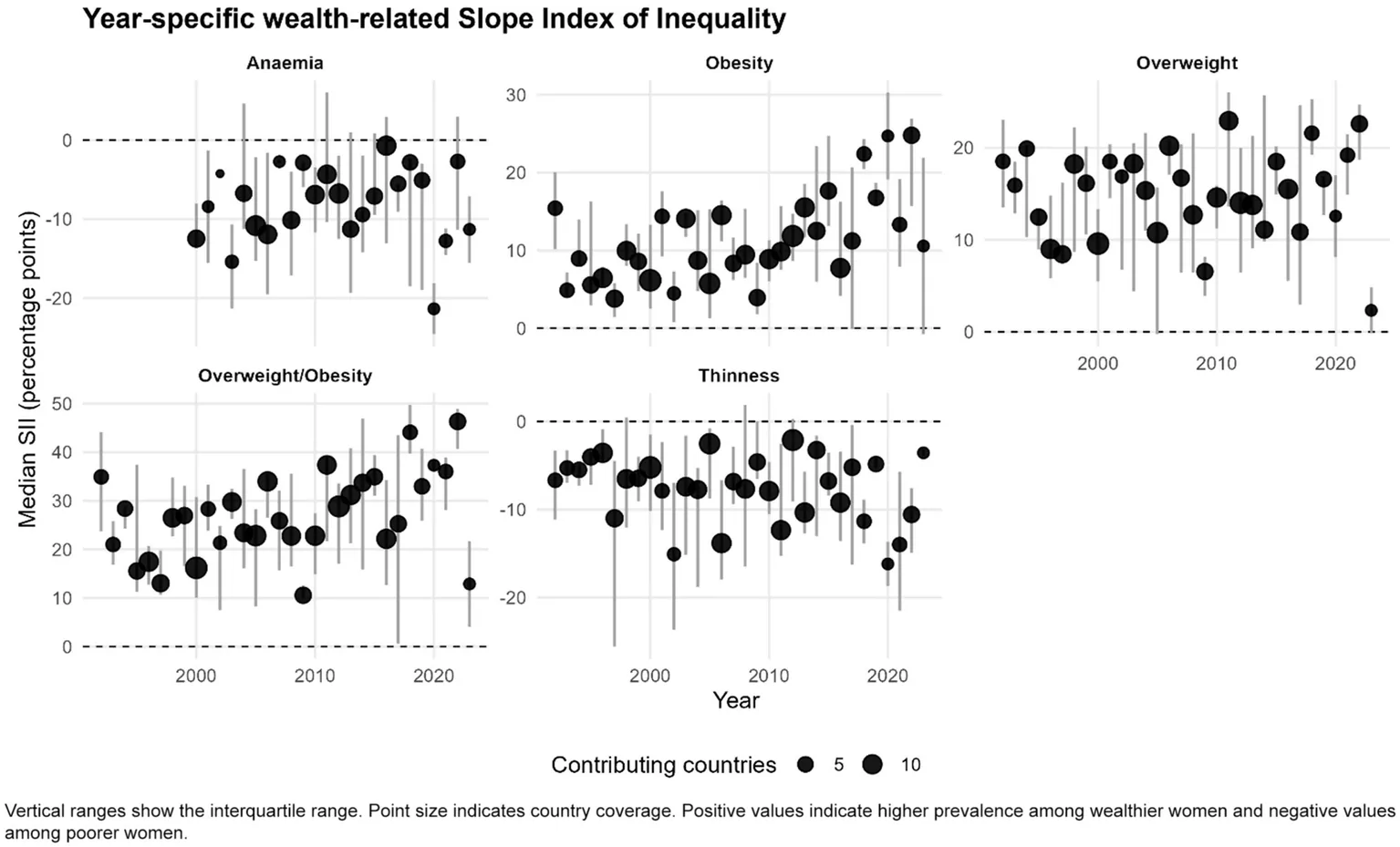
Year-specific wealth-related SII. Points represent unweighted country medians, vertical ranges represent interquartile ranges, and point size indicates the number of contributing countries. Positive SII indicates a higher prevalence among wealthier women, and negative SII indicates a higher prevalence among poorer women. Annual estimates are based on changing country samples.

Wealth-related SII was generally negative for anemia and thinness and positive for overweight, obesity, and combined overweight/obesity. However, year-specific pooled medians were based on different country samples and should not be interpreted as a continuous global trajectory. The number and identity of contributing countries varied considerably, particularly at the beginning and end of the study period (Figure 2).

**Figure 3:**
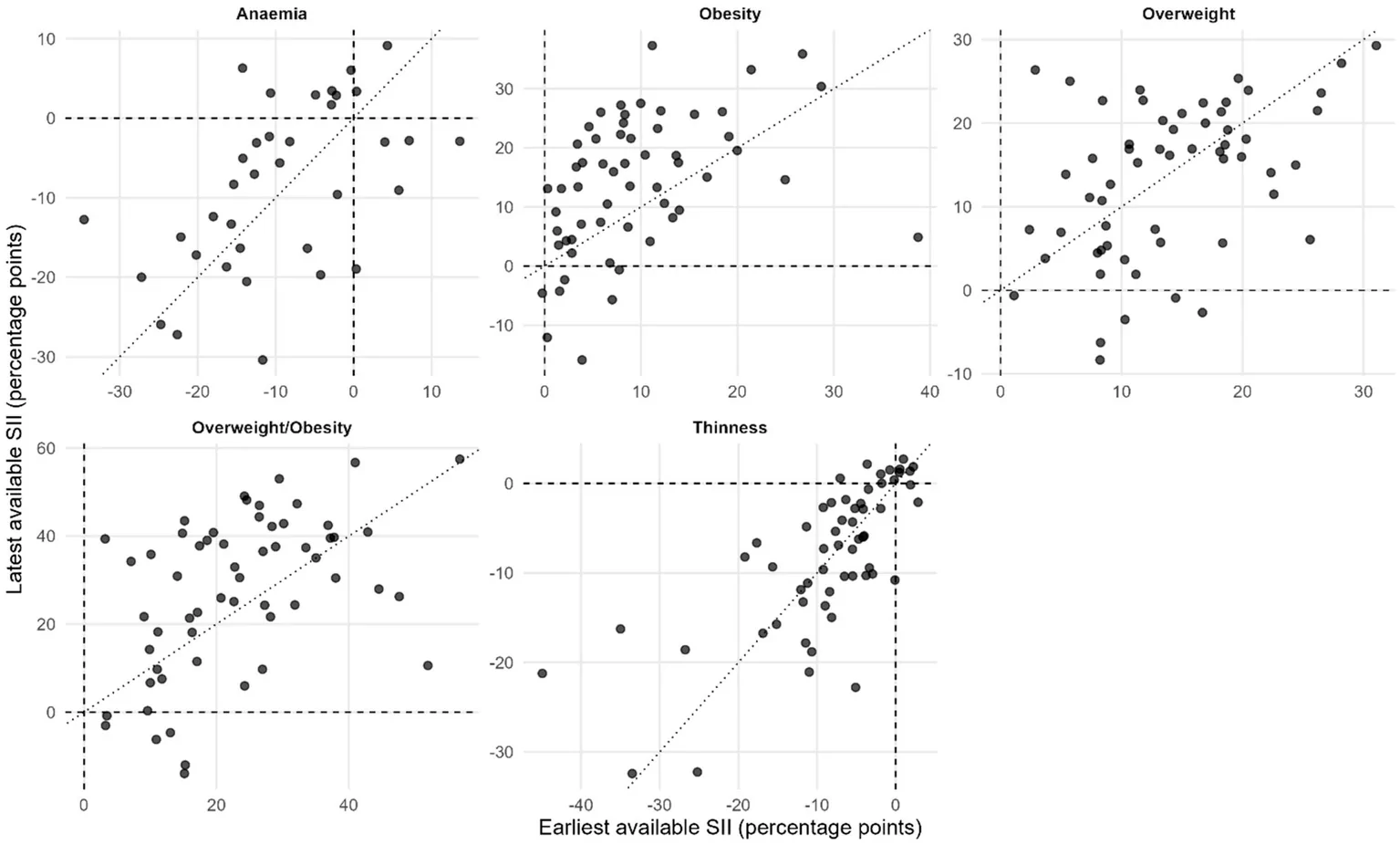
Earliest versus latest wealth-related SII by country. Each point represents one country from at least two surveys. The diagonal line indicates no change in SII; horizontal and vertical zero lines distinguish pro-advantaged from pro-disadvantaged inequality. Differences in follow-up duration and intermediate survey patterns are not represented.

For deficiency-related outcomes, SII values remained predominantly negative across most countries and time points, indicating a persistent concentration of anemia and thinness among disadvantaged groups. Although the magnitude of inequality varied, there was limited evidence of consistent directional change. In contrast, excess-related outcomes showed greater variability in both the direction and magnitude of inequality over time. While overweight and obesity were generally concentrated among more advantaged groups in earlier periods, SII values exhibited wider dispersion in later years, with some countries showing reduced gradients and others approaching neutrality (Figures 2, 3). The distribution of wealth-related SII and extreme-group differences across all available country–year observations is summarized in Supplementary Table S5. The number of countries contributing to each year-specific wealth-SII summary varies considerably over time and is presented in Supplementary Figure S1.

Complete education subgroup panels were available for only 14 of 1,062 eligible observations (1.3%), comprising Armenia in 2000 and 2005 and Kyrgyzstan in 2012; education-related estimates were therefore treated as exploratory and were not interpreted as multi-country temporal trajectories (Supplementary Table S2; Supplementary Figure S5).

### 3.5 Transition patterns in inequality trajectories across countries

To characterize how socioeconomic inequalities evolved over time, countries were classified according to changes in wealth-related SII between earlier and more recent survey periods. Three broad patterns were identified: persistence of the direction of inequality, attenuation of inequality magnitude, and reversal of the socioeconomic gradient.

The direction of excess-related inequality generally remained pro-advantaged. Overweight remained pro-advantaged in 52 of 58 repeated-country series (89.7%), obesity in 49 of 58 (84.5%), and combined overweight/obesity in 51 of 58 (87.9%). Reversal was uncommon, occurring in 6.9% of overweight and obesity series and 8.6% of combined overweight/obesity series. Changes in magnitude were heterogeneous, including attenuation, strengthening, and small changes classified as persistence.

The all-timepoint sensitivity analysis produced more conservative evidence of magnitude change. No clear linear change in absolute SII was found for 95.8% of anemia, 89.5% of thinness, 73.7% of overweight, and 52.6% of combined overweight/obesity series. Obesity was the principal exception, with 50.0% of series showing evidence of strengthening (Table 2; Figure 3). The outcome-specific distribution of the earliest-to-latest transition categories is shown in Supplementary Figure S2, while detailed country-level classifications are provided in Supplementary Table S6.

**Table 2:** Distribution of countries by wealth-related inequality transition patterns.

Outcome	Attenuation	Persistence	Strengthening	Reversal	Emergence/Near-null
Anemia, *n* = 36	11 (30.6%)	6 (16.7%)	6 (16.7%)	10 (27.8%)	3 (8.3%)
Thinness, *n* = 58	19 (32.8%)	12 (20.7%)	18 (31.0%)	3 (5.2%)	6 (10.3%)
Overweight, *n* = 58	15 (25.9%)	18 (31.0%)	21 (36.2%)	4 (6.9%)	0
Obesity, *n* = 58	7 (12.1%)	8 (13.8%)	36 (62.1%)	4 (6.9%)	3 (5.2%)
Overweight/obesity, *n* = 58	10 (17.2%)	14 (24.1%)	29 (50.0%)	5 (8.6%)	0

Transition patterns are based on changes in wealth-related SII between earlier and most recent survey periods. “Pro-disadvantaged” indicates higher prevalence among lower socioeconomic groups (negative SII), while “pro-advantaged” indicates higher prevalence among higher socioeconomic groups (positive SII). Reversal indicates a change in the direction of inequality over time. Country-level transition classifications are provided in Supplementary Table S6.

### 3.6 Model-based synthesis of temporal trends

Mixed-effects models indicated an increasing population-level prevalence of overweight, obesity, and combined overweight/obesity and declining prevalence of thinness across survey years. Anemia showed a comparatively small and imprecise temporal change. All 10 models converged without singular fits. Considerable between-country variability was captured through country-specific random intercepts and time slopes.

**Figure 4:**
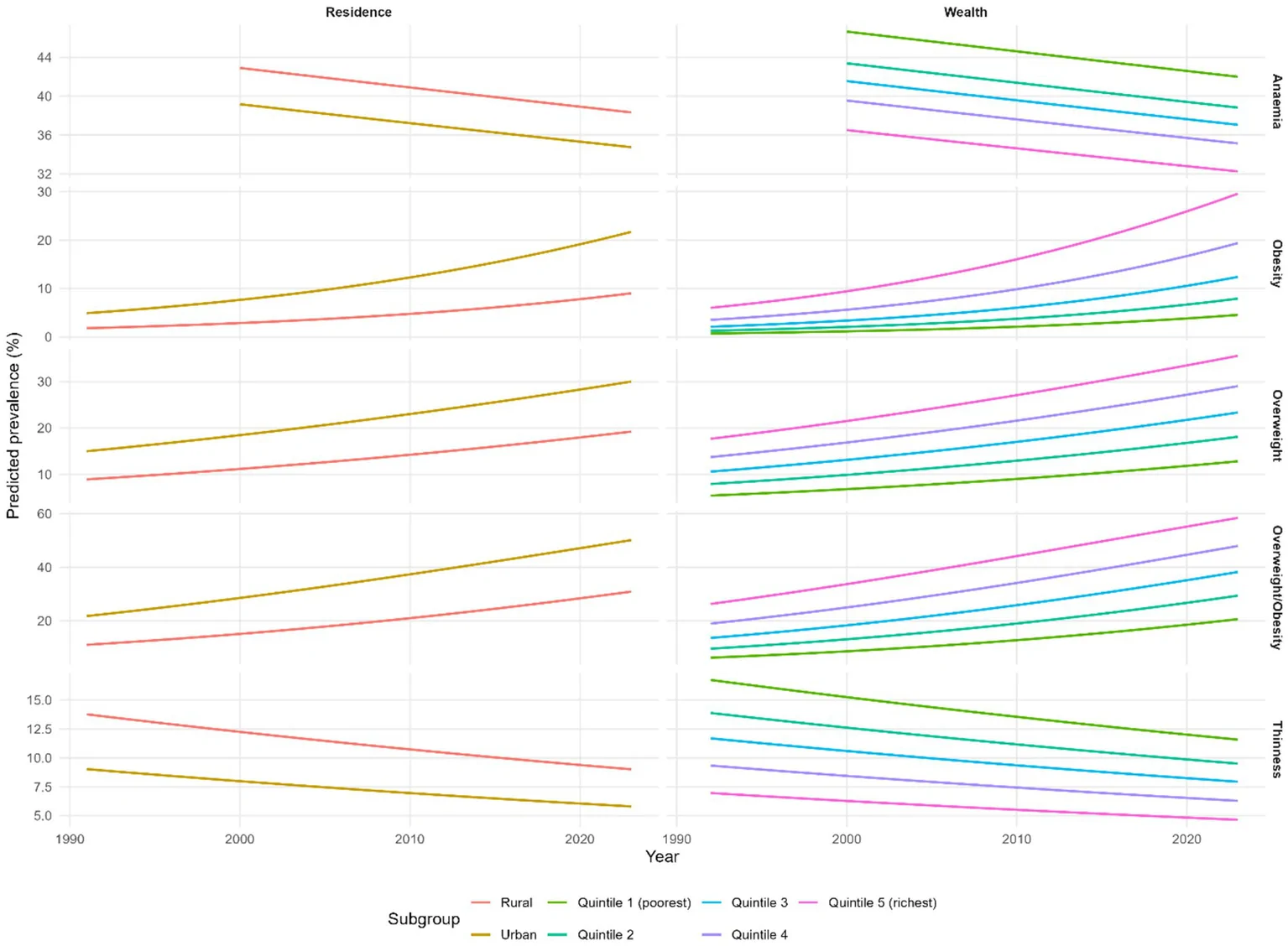
Predicted prevalence trends from multilevel mixed effects models.

These models evaluated subgroup prevalence rather than SII and did not include year-by-subgroup interactions. Consequently, the predicted curves describe overall prevalence trends and average subgroup differences; they do not provide direct evidence that socioeconomic inequality has increased or decreased over time. Country-specific SII changes were evaluated separately in trajectory and sensitivity analyses (Figure 4). The repeated-country and all-timepoint sensitivity analyses are presented in Supplementary Figures S3, S4, respectively.

### 3.7 Cross-country heterogeneity at recent inequality levels

**Figure 5:**
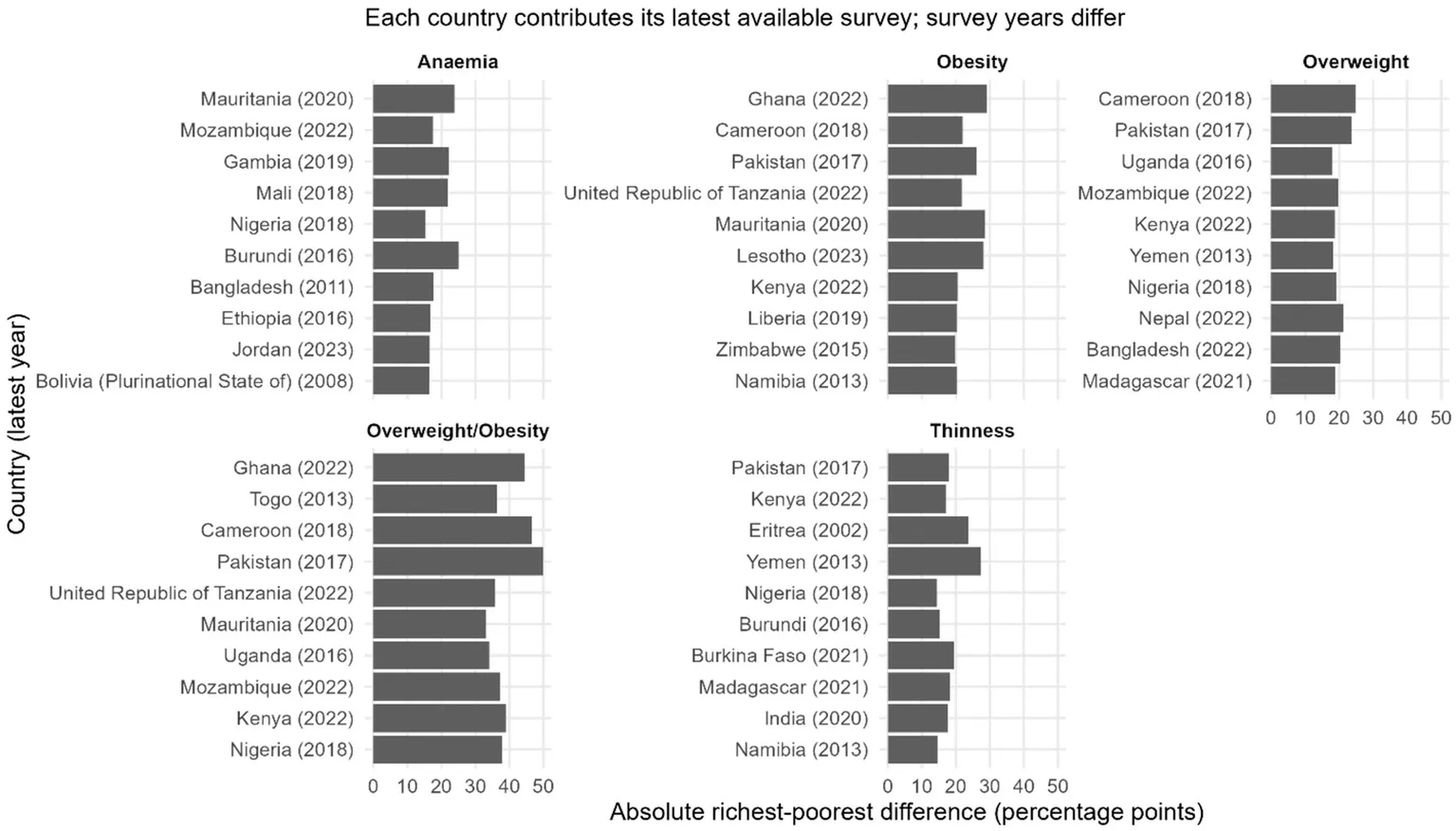
Countries with the largest absolute wealth-quintile prevalence differences at their latest available survey. Bars show the absolute difference in prevalence between the richest and poorest wealth quintiles. Country-specific survey years differ.

Substantial heterogeneity in recent inequality levels was observed across countries (Figure 5; Table 3). In the most recent survey years, the magnitude and direction of wealth-related inequality varied widely between settings, even for the same nutritional outcome. Countries with the largest pro-advantaged inequalities highlighted substantial cross-country variation in both the magnitude and direction of inequality.

**Table 3:** Summary of country-specific latest available prevalence and wealth-related inequality estimates.

Outcome	Countries	Latest-survey years	Median prevalence	Median wealth difference	Median wealth SII
Anemia	56	2000–2023	40.4%	−6.1	−6.22
Thinness	70	1994–2023	7.9%	−4.6	−5.60
Overweight	70	1994–2023	22.2%	+11.2	+13.98
Obesity	70	1994–2023	11.7%	+10.5	+13.21
Overweight/obesity	70	1994–2023	33.7%	+20.8	+25.02

Each country contributed to the latest available survey. Survey years differed across countries and ranged from 2000 to 2023 for anemia and 1994–2023 for BMI outcomes; the median country-specific latest survey year was 2016. These estimates are descriptive and should not be interpreted as a common-period comparison.

For deficiency-related outcomes, most countries continued to show negative SII values, indicating a concentration of anemia and thinness among disadvantaged populations. However, the extent of inequality across countries varied widely, from modest gradients to pronounced disparities. Excess-related outcomes showed greater variability in both magnitude and direction. Instead, some countries retained positive SII values, consistent with concentration among more advantaged groups; others showed weaker gradients or values closer to zero, indicating a more even distribution across socioeconomic groups. Countries with the largest absolute inequalities, whether positive or negative, highlight the extent to which social patterning of nutritional outcomes differs across settings. These findings reinforce that average global patterns mask substantial cross-country variation in both the level and direction of inequality.

## 4 Discussion

It was found that socioeconomic inequalities in women’s malnutrition were predominantly persistent in direction but heterogeneous in magnitude across countries. Anemia and thinness generally stayed concentrated among disadvantaged women, whereas overweight and obesity generally remained concentrated among advantaged women. Reversal occurred in a relatively small minority of countries. Sensitivity analyses using all available survey rounds found no clear linear change in magnitude for most countries’ series, although strengthening was more frequent for obesity. While some patterns remain stable, particularly for deficiency-related outcomes, others appear to be shifting, and not always in the same direction across countries. In conclusion, it could be stated that inequality patterns did not appear to converge across countries; instead, persistence, attenuation, and reversal were observed simultaneously across different contexts. Heterogeneousness is consistent with prior studies emphasizing that nutritional and epidemiological transitions do not expand uniformly across populations (6, 27). Model-based analyses further reinforced these patterns, indicating that while average trends were consistent, substantial between-country variability remained.

Importantly, these findings suggest that changes in overall prevalence do not necessarily correspond to reductions in inequality. In several settings, improvements in nutritional indicators appear to have occurred alongside persistent or only modestly changing socioeconomic gradients, underscoring the distinction between population-level progress and equity in its distribution. At the same time, the observed variability in excess-related outcomes, including signs of attenuation or reversal in some countries, points toward a heterogeneous and context-specific pattern of inequality than is often assumed.

Education-based estimates were available for only 14 complete panels from Armenia and Kyrgyzstan and cannot be generalized across LMICs, regions, or survey periods. These estimates are presented only as an exploratory coverage analysis and are not used to support the main conclusions. A clear distinction emerged between deficiency- and excess-related outcomes. Anemia and thinness remained consistently concentrated among disadvantaged populations, with relatively stable inequality patterns over time, consistent with prior evidence highlighting the persistent social gradient of undernutrition in LMICs (3, 4). This persistence suggests that, despite overall improvements in some settings, reductions in average prevalence have not translated into meaningful reductions in socioeconomic inequality. In that sense, the persistence of disadvantage-associated deficiency is consistent with the influence of broader social and structural conditions, although these mechanisms were not directly examined.

Overweight and obesity generally remained concentrated among advantaged women, although their magnitude varied and a small number of countries showed reversal. These patterns may be consistent with countries being at different stages of nutrition transition, but the present analysis did not directly examine food environments, urbanization, dietary changes, physical activity, or other mechanisms. Nutrition transition should therefore be presented as a plausible interpretation supported by previous literature rather than as an empirically demonstrated explanation in the present analysis (8, 9). These patterns are consistent with variation in the social distribution of excess weight across countries and survey periods in response to broader contextual changes but not universally. These findings point to a more complex picture than a simple or linear “nutrition transition.” Rather than moving uniformly from undernutrition to overnutrition, countries appear to experience overlapping and sometimes contradictory patterns, reflecting the multidimensional nature of nutritional change observed across LMICs (3, 27). In some contexts, excess burden increases without substantial reductions in deficiency, while in others, the social distribution of risk begins to shift. The coexistence of persistent disadvantage-associated nutritional deficiency and evolving excess-burden inequality suggests that multiple transitions may occur simultaneously, rather than sequentially.

Another feature that stood out, perhaps more than expected, was the degree of heterogeneity across countries. Even among countries with broadly similar economic classifications, inequality trajectories differed in both magnitude and direction. This variation suggests that macro-level indicators alone may not fully explain how nutritional inequalities evolve, consistent with prior studies highlighting the importance of context-specific determinants and policy environments (15, 16). Instead, context-specific factors, such as food environments, urbanization, health systems, and policy responses, may play a role, although these could not be examined directly in the present analysis.

From a policy perspective, these findings highlight the limitations of one-dimensional approaches to malnutrition. Efforts to reduce undernutrition remain essential, particularly for disadvantaged populations where deficiency remains concentrated, consistent with long-standing evidence on the social distribution of undernutrition (3). At the same time, strategies addressing overweight and obesity may need to account for heterogeneous socioeconomic distributions, rather than assuming that the excess burden is confined to more advantaged groups.

These findings align with growing recognition that the “double burden” of malnutrition requires integrated and context-specific policy responses that address both deficiency and excess simultaneously (6, 28). In settings where socioeconomic gradients are weakening or reversing, prevention strategies may need to move beyond narrowly targeted approaches and adopt broader population-level interventions responsive to changing patterns of risk.

This study has several limitations. First, the analysis used aggregated repeated cross-sectional estimates and cannot establish individual-level longitudinal change or causal mechanisms. Second, survey years and the number and spacing of survey rounds differed across countries. Annual pooled medians were therefore based on changing country samples, and countries with more surveys contributed observations to more calendar years. Third, earliest-to-latest classifications may be sensitive to endpoint fluctuations and unequal follow-up duration. Although an all-timepoint sensitivity analysis was undertaken, many country series contained only three observations and statistical power was limited. Fourth, analyses were restricted to complete country-year subgroup panels to ensure internally comparable inequality estimates. Because the WHO Health Inequality Data Repository provides aggregated subgroup estimates rather than individual-level data, a formal comparison between included and excluded observations was not feasible. Consequently, some potential for selection bias cannot be excluded, although the completeness criteria were applied consistently across all countries, survey years, and nutritional outcomes. Fifth, education coverage was restricted to two countries and was not representative of LMICs. Sixth, the subgroup-level data did not permit adjustment for individual-level confounding or complex within-group heterogeneity. Finally, the analyses describe changes in prevalence and inequality but do not directly evaluate the mechanisms underlying those changes. Despite these limitations, the study provides a broad and internally consistent assessment of socioeconomic inequalities in women’s nutrition using harmonized repeated cross-sectional data. It jointly describes the national prevalence, the magnitude and direction of inequality, and country-specific transition patterns while explicitly accounting for unbalanced survey timing.

## 5 Conclusion

Socioeconomic inequalities in women’s malnutrition across LMICs were predominantly persistent in direction but heterogeneous in magnitude. Anemia and thinness generally remained concentrated among disadvantaged women, whereas overweight and obesity generally remained concentrated among advantaged women. Reversal was uncommon, and sensitivity analyses using all survey rounds found no clear linear change in magnitude for most outcomes, although obesity inequalities were more frequently strengthened. These findings emphasize the need to monitor national prevalence and socioeconomic inequality separately and to interpret cross-country differences in relation to each country’s survey coverage and context.

## Data Availability

The original contributions presented in the study are included in the article/Supplementary material, further inquiries can be directed to the corresponding authors.
