# Supplementary material for: Persistent and heterogeneous socioeconomic inequalities in women’s malnutrition across 70 low- and middle-income countries
Source: Front Public Health. 2026 Aug 20;14:1910404. doi: 10.3389/fpubh.2026.1910404 (PMC13538111; doi:10.3389/fpubh.2026.1910404)
Supplement: Supplementary file 1 [file Data_Sheet_1.ZIP › supplementay materials/Supplementary materials.docx]

**Supplementary Table S1: Structure of inequality dimensions and subgroup ordering**

| Dimension | Dimension (short) | Subgroup | Order | Reference group | Ordered dimension |
| --- | --- | --- | --- | --- | --- |
| Education (2 groups) | Education | No or primary education | 1 | 0 | No |
| Education (2 groups) | Education | Secondary or higher education | 2 | 1 | No |
| Education (4 groups) | Education | No education | 1 | 0 | Yes |
| Education (4 groups) | Education | Primary education | 2 | 0 | Yes |
| Education (4 groups) | Education | Secondary education | 3 | 0 | Yes |
| Education (4 groups) | Education | Higher education | 4 | 0 | Yes |
| Place of residence | Residence | Rural | 1 | 0 | No |
| Place of residence | Residence | Urban | 2 | 1 | No |
| Economic status | Wealth | Quintile 1 (poorest) | 1 | 0 | Yes |
| Economic status | Wealth | Quintile 2 | 2 | 0 | Yes |
| Economic status | Wealth | Quintile 3 | 3 | 0 | Yes |
| Economic status | Wealth | Quintile 4 | 4 | 0 | Yes |
| Economic status | Wealth | Quintile 5 (richest) | 5 | 0 | Yes |

Subgroups are ordered from most disadvantaged to most advantaged for ordered dimensions (wealth and education with four categories). Binary dimensions (education with two groups and place of residence) are treated as non-ordered contrasts. Reference groups are indicated for pairwise comparisons. Ordered dimensions were used to construct relative socioeconomic rank variables for regression-based inequality measures (SII).

**Supplementary Table S2: Subgroup completeness across inequality dimensions**

| Dimension | Candidate panels | Complete panels | Completeness | Countries | Years |
| --- | --- | --- | --- | --- | --- |
| Wealth | 1,038 | 1,038 | 100.0% | 70 | 1992–2023 |
| Residence | 1,042 | 1,030 | 98.85% | 70 | 1991–2023 |
| Education | 1,062 | 14 | 1.32% | 2 | 2000–2012 |

**Supplementary Table S3. Included countries and historical income-group (given as separate excel sheet)**

**Supplementary Table S4: List of available indicators and selection criteria**

| Indicator | Indicator abbreviation | Included in analysis | Reason |
| --- | --- | --- | --- |
| Anaemia prevalence (%) – Female | AN_ANEM_ANY_F | Yes | Core outcome |
| Thinness prevalence (BMI <18.5 kg/m²) (%) – Female | AN_NUTS_THN_F | Yes | Core outcome |
| Overweight prevalence (BMI 25.0–29.9 kg/m²) (%) – Female | AN_NUTS_OVW_F | Yes | Core outcome |
| Obesity prevalence (BMI ≥30.0 kg/m²) (%) – Female | AN_NUTS_OBS_F | Yes | Core outcome |
| Overweight or obesity prevalence (BMI ≥25.0 kg/m²) (%) – Female | AN_NUTS_OWT_F | Yes | Core outcome |
| Severe anaemia prevalence (%) – Female | AN_ANEM_SEV_F | No | Limited availability |
| Height <145 cm (%) – Female | AN_NUTS_SHT_F | No | Not central to the study aim |
| Smoking prevalence (%) – Female | AH_TOBC_ANY_F | No | Non-nutritional indicator |

Five nutritional outcomes were included based on their relevance to deficiency- and excess-related malnutrition and the availability of sufficiently complete country–year subgroup data.

**Supplementary Table S5: Distribution of inequality measures across all country year observations**

| Outcome | Burden | n | Median SII | Mean SII | Range | Median absolute difference | Mean absolute difference |
| --- | --- | --- | --- | --- | --- | --- | --- |
| Anemia | Deficiency | 133 | −6.89 | −7.79 | −34.58 to 20.70 | 7.0 | 8.7 |
| Thinness | Deficiency | 227 | −6.64 | −8.72 | −44.93 to 4.43 | 5.2 | 7.6 |
| Overweight | Excess | 226 | 14.99 | 13.98 | −8.33 to 33.15 | 11.6 | 11.7 |
| Obesity | Excess | 226 | 10.92 | 11.44 | −15.89 to 38.74 | 9.4 | 10.0 |
| Overweight or obesity | Excess | 226 | 26.48 | 25.41 | −13.97 to 60.14 | 21.4 | 21.3 |

Values summarize inequality measures across all country year observations with complete subgroup data. SII (Slope Index of Inequality) reflects absolute inequality across the full socioeconomic gradient, with negative values indicating concentration among disadvantaged groups and positive values indicating concentration among advantaged groups. Absolute differences represent differences between extreme subgroups.

**Supplementary Table S6: Country-level earliest to latest wealth-related inequality transitions (given as separate excel sheet)**

**Figure S1: Number of countries contributing to annual wealth-SII summaries**

**Figure S2: Earliest-to-latest wealth-SII change categories**

**Figure S3: Sensitivity analysis among countries with repeated observations**

**Figure S4: All-timepoint wealth-SII sensitivity analysis**

**Figure S5: Coverage of exploratory education-related inequality estimates. Complete education panels were available only for Armenia and Kyrgyzstan during 2000–2012. Education results were therefore not interpreted as multi-country temporal trajectories.** Finally, these temporal patterns suggest that while inequality in deficiency related outcomes remains relatively stable, the distribution of excess-related outcomes is more variable and potentially evolving across settings.

**Supplementary File S1. Reproducible analytical R script**

**File name:** WHO_women_nutrition_inequality_reviewer_revision_2026-07-24.R

This script contains the complete analytical workflow used in this study. Specifically, it implements:

- Import of the WHO Health Inequality Data Repository (Adult Health and Nutrition; DHS) dataset.
- Data cleaning, harmonization, and variable standardization.
- Historical LMIC eligibility assessment.
- Subgroup-completeness evaluation.
- Construction of the analytical dataset.
- Population-weighted Slope Index of Inequality (SII) estimation.
- Earliest-to-latest trajectory classification.
- Sensitivity analyses using all available survey rounds.
- Population-weighted mixed-effects modelling.
- Generation of all tables, figures, quality-control outputs, and supplementary results reported in the manuscript.

To reproduce the analyses, users should update the input and output directory paths as required for their local computer before running the script.
